# On cross-correlations, averages and noise in electron microscopy

**DOI:** 10.1107/S2053230X18014036

**Published:** 2019-01-01

**Authors:** Michael Radermacher, Teresa Ruiz

**Affiliations:** a Department of Molecular Physiology and Biophysics, University of Vermont, 149 Beaumont Avenue, Burlington, VT 05405, USA

**Keywords:** image processing, signal-to-noise ratio, cross-correlation, multireference alignment, 3D reference-based projection alignment

## Abstract

Biological samples are radiation-sensitive and require imaging under low-dose conditions to minimize damage. As a result, images contain a high level of noise and exhibit signal-to-noise ratios that are typically significantly smaller than 1. Averaging techniques, either implicit or explicit, are used to overcome the limitations imposed by the high level of noise. Averaging of 2D images showing the same molecule in the same orientation results in highly significant projections. A high-resolution structure can be obtained by combining the information from many single-particle images to determine a 3D structure. Similarly, averaging of multiple copies of macromolecular assembly subvolumes extracted from tomographic reconstructions can lead to a virtually noise-free high-resolution structure. Cross-correlation methods are often used in the alignment and classification steps of averaging processes for both 2D images and 3D volumes. However, the high noise level can bias alignment and certain classification results. While other approaches may be implicitly affected, sensitivity to noise is most apparent in multireference alignments, 3D reference-based projection alignments and projection-based volume alignments. Here, the influence of the image signal-to-noise ratio on the value of the cross-correlation coefficient is analyzed and a method for compensating for this effect is provided.

## Abstract 2

The influence of noise on cross-correlations is revisited. Equations are provided describing the influence of noise on the cross-correlations between single images and averaged images and on those between averaged images.

## 1. Introduction   

Cryo-electron microscopy of biological samples has made large strides towards achieving close to atomic resolution structure determination (Cheng *et al.*, 2017 ▸; Nobel Foundation, 2017 ▸). Since biological samples are radiation-sensitive, micrographs are recorded under low-dose conditions, resulting in images with signal-to-noise ratios (SNRs) substantially lower than 1 (or negative decibels; dB). In contrast, the detection of image details by the human eye requires SNRs with values ranging between 4 and 5 (Rose, 1973 ▸). To overcome this problem in biological electron microscopy, images of single particles are aligned, classified and averaged to obtain clear projection images. 3D reconstructions are calculated from many images showing the molecule or macromolecular assembly in multiple orientations, using a much larger number than would be required by the sampling conditions. For 2D averaging, images are aligned rotationally and translationally, classified with multivariate statistical methods combined with multireference alignments, and subsequently the images corresponding to each class are averaged separately (Frank, 1975 ▸, 1978 ▸; Frank *et al.*, 1978 ▸; van Heel & Frank, 1981 ▸; van Heel & Stöffler-Meilicke, 1985 ▸). For 3D reconstructions a number of methods are used, all of which include a variation of a cross-correlation process. If tomographic reconstructions are used as a starting point, they are often followed by 3D alignments and averaging. Cross-correlations are also present when angular reconstitution or random conical tilt methods are applied to obtain first references, or when random conical tilt methods are applied for resolving multiple structures representing different conformations of a highly heterogeneous sample (Radermacher *et al.*, 1987 ▸; van Heel, 1987 ▸; Radermacher, 1988 ▸; Bartesaghi & Subramaniam, 2009 ▸; Yu *et al.*, 2010 ▸, 2013 ▸; Schmid, 2011 ▸; Asano *et al.*, 2016 ▸; Wan & Briggs, 2016 ▸). Most of these techniques are followed by 3D reference-based projection alignments, in which the projection angles and *xy* positions are refined using cross-correlation methods between a 3D reference structure and single 2D projections, or in which additional 2D projections are first aligned with a 3D reference and subsequently added to the 3D reconstruction (see, for example, Radermacher & Ruiz, 2006 ▸; Scheres *et al.*, 2007 ▸).

All of the above averaging approaches either explicitly or implicitly use cross-correlation methods, and the very low SNR of the data may adversely affect the image processing and bias the results. The effect of noise on the cross-correlation coefficient has been described previously for correlations between two images with the same SNR (Bershad & Rockmore, 1974 ▸; Frank & Al-Ali, 1975 ▸). Many steps in the processing of single-particle data sets, however, include cross-correlation procedures of images with different SNRs. These include, but are not limited to, the correlation of a single 2D image with a 2D average image, or the correlation of a 2D projection with a 3D volume reconstructed from a 2D projection set that is not evenly distributed, thus exhibiting different SNRs in different directions, which are apparent along the radial lines of the polar 3D Fourier transform or the 3D Radon transform. Since 3D projection alignments utilize comparisons of the projection transform with the central sections of the 3D transform of the structure, the varying SNR may bias the alignment results.

In 2D and 3D multireference alignment procedures, cross-correlation coefficients are explicitly used when deciding the assignment of a test image or volume to a specific reference. Here, we analyze the effect of noise on the value of the cross-correlation coefficient in 2D and 3D applications.

## 2. Theory   

The value of the cross-correlation maximum, when two images are cross-correlated, depends on the agreement between the motifs in each image and on their SNR. In electron microscopy image processing all 2D images and 3D structures typically originate from projections with similar noise content. This allows the calculation of the influence of noise on the cross-correlation coefficient not only when correlating two images with the same SNR, but also when correlating single images to an average image from the same data set, or when correlating two averages, again derived from the same data set. The following calculations are estimates and use approximations. They will aid, however, in judging the effect of variations in the SNR on the outcome of a calculation. For simplicity we use the following assumptions: (i) the noise is white, additive and Gaussian-distributed with an average equal to 0, (ii) the signal and the noise are uncorrelated and (iii) the average of the signal is 0. The latter assumption is used to simplify the calculations but does not affect the results.

From assumption (i) it follows that the expectation value of the noise cross-correlation is 0 and
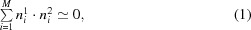
where *n_i_* ^1^ and *n_i_* ^2^ are two independent realizations of Gaussian-distributed white noise and *M* is the number of pixels in an image.

From assumption (ii), stating that the signal and noise are uncorrelated, it follows that the cross-correlation between signal and noise also vanishes,
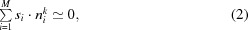
where *s_i_* is the signal and *n_i_* ^*k*^ is the *k*th realization of Gaussian-distributed white noise.

In the following, the SNR α is defined as the ratio of the variances:
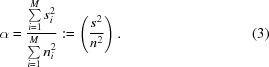
Under these assumptions, the well known equation for the value of the cross-correlation in the presence of noise can be derived (Bershad & Rockmore, 1974 ▸; Frank & Al-Ali, 1975 ▸). Let *C* be the normalized cross-correlation coefficient and (*s_i_* + *n_i_^x^*) and (*s_i_* + *n_i_^y^*) two images with the same motif but different noise. When no noise is present, the cross-correlation coefficient *C* is 1.
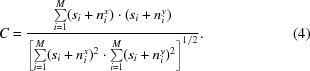
Developing this equation, we obtain
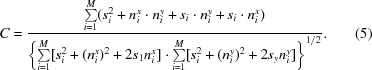
Using the approximations stated above, the mixed signal and noise terms yield 0, the cross-correlation of noise terms yield 0, and since *n_i_^x^* and *n_i_^y^* are two realizations of Gaussian distributed white noise 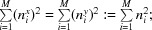
thus, (5) simplifies to
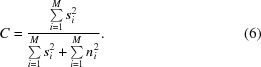
Defining 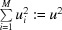
and using the definition of the SNR in (3), α = (*s* ^2^/*n* ^2^), which allows the substitution of *s* ^2^ by *s* ^2^ = α · *n* ^2^, (6) yields
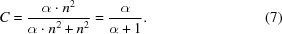
The relation described in (7) can be used to determine the SNR of a series of images by calculating their pairwise cross-correlations (Frank & Al-Ali, 1975 ▸):
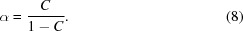
(7) describes the cross-correlation coefficient of two images with the same SNR. However, more often cross-correlations are used for the alignment of noisy images with an average reference with a larger SNR. If the reference represents an average of images with the same signal and with the same SNR, then the cross-correlation is also noise-dependent and has a value smaller than 1. The cross-correlation coefficient depends on the SNR of each single image and on the number of images used to calculate the average image.

It is well known that averaging improves the SNR. When averaging *N* images the standard deviation of the noise is reduced by *N* ^1/2^. Thus, if α is the SNR of a single image, defined as above as the ratio of signal and noise variances, the SNR of an average image containing *N* images is β = α · *N*. The cross-correlation between a single image and the average image can then be calculated.

From (3), the SNR of a single image is α = (*s* ^2^/*n* ^2^) and the SNR of the averaged image results in
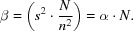
Defining the superscript *x* in (4) to indicate the single image and the superscript *y* to indicate the average, and using 
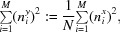
(5) becomes
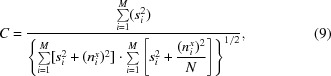
and substituting *s* by the SNR α using the formula 
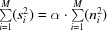
in (9) yields
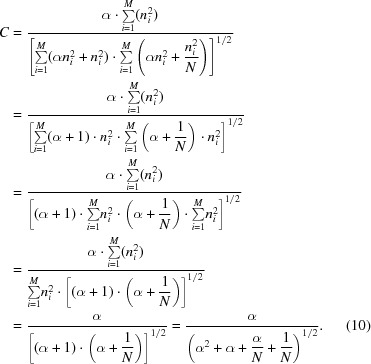
The cross-correlation coefficient *C* in (10) represents the value obtained when a single image is cross-correlated with an average image containing *N* images of the same image set with the same SNR.

Equation (10) can be used to determine the SNR from the cross-correlation of single images to an average image obtained fom the same data set:
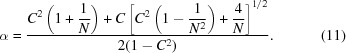
For *N* = 1, (11) simplifies to (8) for determining the SNR from cross-correlations of single images.

The calculations can easily be extended to the cross-correlation between two average images calculated from different numbers of images. We can assume that both images are average images of the single images with noise *n^x^*, the first average image contains *L* single images and the second average image contains *N* single images. (5) can then be rewritten as
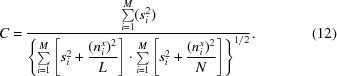
Performing the same calculations as above and substituting *s* by the SNR α using the equation 
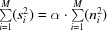
yields
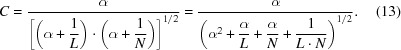
The cross-correlation coefficient *C* in (13) represents the value obtained when an average image containing *L* single images is cross-correlated with an average image containing *N* single images of the same image set with the same SNR per image.

## 3. Test calculations and a cryo-EM data example   

**Figure 1:**
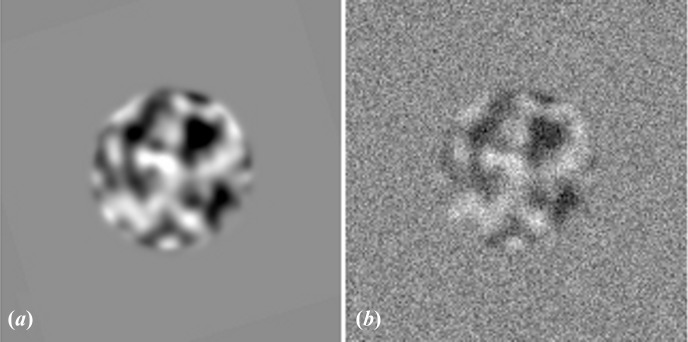
Test data. (*a*) Motif, (*b*) one of the 400 images with added noise with SNR = 0.5.

The theory was initially tested with model data for both pairwise correlations and correlations towards an average. We created model images with an SNR of 0.5 (Fig. 1 ▸). The motif was generated by first creating a random noise image, low-pass filtering and thresholding it to reintroduce higher frequencies; it was then rotated and masked. To introduce noise, a large image containing Gaussian-distributed white noise was first created. Subsequently, rows of non-overlapping images with the same size as the motif were boxed out of the large noise image and the motif was added to obtain the final images. Both the motif and the noise image were scaled such that the variances of signal to noise had a ratio of 0.5. A total of 400 images were created. In a first experiment, all 400 images were cross-correlated in unique pairs, excluding auto-correlations. The average cross-correlation coefficient was determined to be 0.334, with a standard deviation of 0.0084. The SNR for each cross-correlation coefficient, calculated using (8), yielded an average SNR of 0.501 with a standard deviation of 0.011. In a second experiment 50 images were averaged. Each of the 400 single images was cross-correlated with the average and yielded normalized cross-correlation coefficients with an average of 0.557 and a standard deviation of 0.007. The resulting SNR calculated as an average of individual SNRs using (11) was 0.508 with a standard deviation of 0.016, which is in good agreement with the SNR determined by pairwise correlation. All calculations were carried out using the *Environment for Modular Image Reconstruction Algorithms* (*EMIRA*; Radermacher, 2013 ▸).

**Figure 2:**
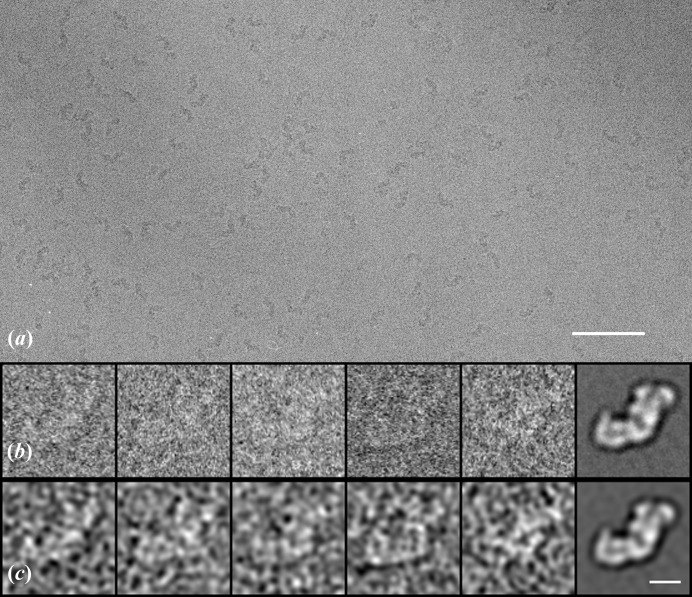
Cryo-electron microscopy data for complex I from *Y. lipolytica*. (*a*) Area of micrograph. (*b*) Selection of boxed-out images and average image. (*c*) The same images as in (*b*), low-pass filtered. The scale bar is 100 Å in length.

The theory was subsequently applied to a set of experimental images for both pairwise correlations and correlations with an average. The data set used contained images of mitochondrial complex I purified from the yeast *Yarrowia lipolytica* (Radermacher *et al.*, 2006 ▸) and prepared for microscopy in vitreous ice (Fig. 2 ▸ *a*). Images were recorded on an FEI Tecnai 12 electron microscope equipped with an LaB_6_ filament operated in point mode at 100 kV (Ruiz *et al.*, 2003 ▸; Ruiz & Radermacher, 2006 ▸). The typical defocus was approximately 1.8 µm and the nominal magnification was 52 000×. The micrographs were digitized on an SCAI flatbed scanner with a pixel size of 7 µm and subsequently binned down by a factor of three, resulting in a calibrated pixel size of 4 Å. A total of 1750 images of single particles were boxed out and subjected to multiple rounds of alignment, correspondence analysis and classification using Diday’s method of moving centers (Diday, 1971 ▸), and ten final classes were obtained. Calculations to test the theory presented in this paper were carried out using the class with the largest number of particles, class 8, which contained 462 images (Figs. 2 ▸ *b* and 2 ▸ *c*). The original images had dimensions of 160 × 160; however, for this cross-correlation experiment the images were boxed down to 80 × 80 to exclude most of the image background areas. No additional mask was applied, since the cross-correlation program used normalizes over the whole image and does not allow normalization restricted to a specified mask. All images were low-pass filtered to 33 Å, the resolution of the average image determined by Fourier ring correlation with a cutoff of 0.5.

SNRs were determined using the same approach as for the model data for both pairwise correlations and correlations to an average. First all 462 images were cross-correlated pairwise (using only unique pairs and excluding autocorrelations) and SNR values were calculated from each cross-correlation coefficient. For pairwise cross-correlation the average cross-correlation value was 0.279 with a standard deviation of 0.053 and the averaged SNR was 0.394 with a standard deviation of 0.104. When the average of all 462 images was used as a reference, the average cross-correlation coefficient was 0.530 with a standard deviation of 0.047 and the resulting averaged SNR was 0.404 with a standard deviation of 0.099. The SNR value found relative to the average is slightly higher, since in the cross-correlation of individual images variations in the motif are expected to lower the average cross-correlation coefficients.

**Figure 3:**
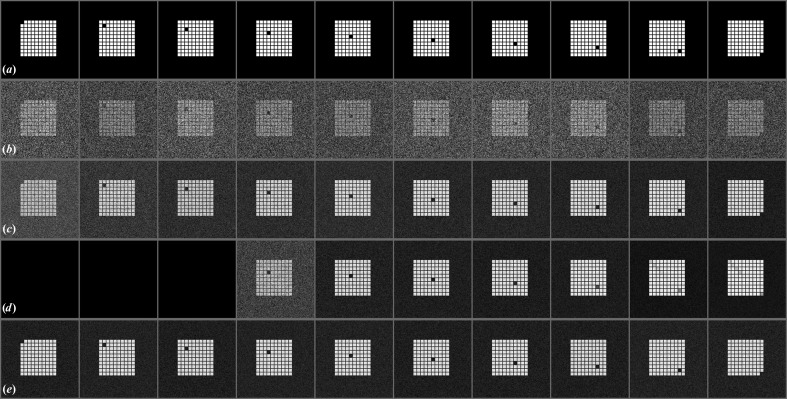
Compensation for the negative effect of the SNR on the cross-correlation. (*a*) The ten motifs used in model calculation. (*b*) Example images of the ten motifs with added noise, SNR = 0.5. (*c*) Averages used as references calculated from 10, 20, 30, 40, 50, 60, 70, 80, 90 and 100 images (from left to right). (*d*) Result of a classification using conventional multireference correlation without correction. (*e*) Result of a classification using multireference correlation with the correction described in (10) implemented.

The negative effect of the SNR on the cross-correlation can be compensated for. We generated a data set containing 1000 images with an SNR of 0.5. The signal image was a pattern of 99 squares, and for each of the ten motifs created a different square was missing (Fig. 3 ▸ *a*). The final data set contained 100 copies of each motif with noise added as before (Fig. 3 ▸ *b*). Ten references for cross-correlation were calculated, the first one by averaging ten images containing the first motif, the second by averaging 20 images containing the second motif *etc.*, until the tenth containing the tenth motif was calculated by averaging 100 images (Fig. 3 ▸ *c*). The 1000 images were recreated using a different noise image to avoid any noise correlation between the averages and single images. The images were correlated in a multireference (translational) alignment using a normalized cross-correlation. During this test no filters or masks were applied since this would have changed the SNR of the data. In a conventional multireference correlation, no images were assigned to averages 1–3 and the plurality of images were assigned to reference 10 (Fig. 3 ▸ *d*). The multireference alignment process was repeated using the inverse of (10) to correct the cross-correlation values. After applying the correction the procedure assigned each image to its correct reference (Fig. 3 ▸ *e*, Table 1 ▸).

## 4. Discussion   

The calculations show the strong dependence of cross-correlation coefficients on the image noise.

**Figure 4:**
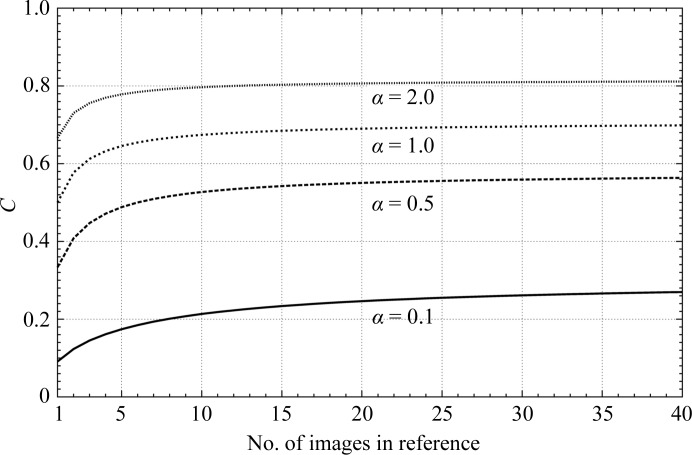
Value of the cross-correlation coefficient between an image with SNR = α and an average image, depending on the number of images averaged in the reference image. Curves are shown for four different SNR values. Abscissa, number of images used to calculate the reference average image. Ordinate, cross-correlation coefficient *C*.

Equation (10) describes the value of the cross-correlation coefficient when a single image with SNR = α is correlated with an average image calculated from *N* single images with the same SNR. In cross-correlation alignment processes the cross-correlation coefficient corresponds to the maximum of the correctly normalized cross-correlation function. While the specific value of the cross-correlation is of minor importance during rotational and translational alignments against a single reference, it can bias the results in multireference alignments when the references are averages of only a few and different numbers of images (van Heel & Stöffler-Meilicke, 1985 ▸). In this situation the cross-correlation with the reference with the highest number of images averaged will show a higher valued cross-correlation, and if used in an iterative procedure, images may tend to be assigned to the reference that starts with the highest number of images averaged. Fig. 4 ▸ illustrates the influence of the number of images used to calculate the reference on the cross-correlation coefficient. While for an SNR of >0.5 the change in the cross-correlation is minimal when more than 40 images are averaged, for lower values of the SNR the dependence on the number of images averaged to calculate the reference is still strong.

**Figure 5:**
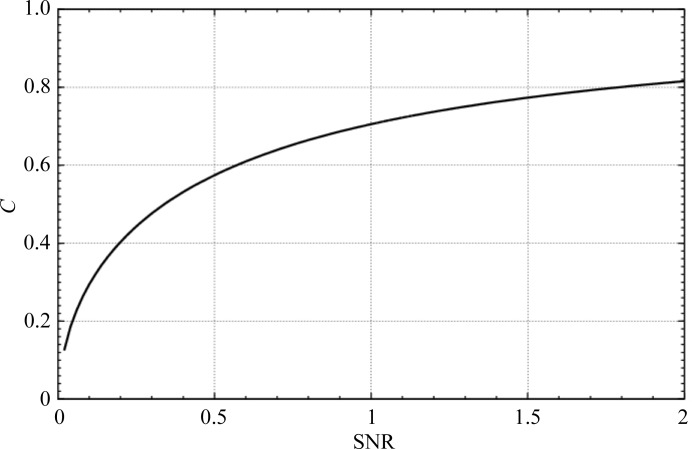
Asymptotic value of the cross-correlation coefficient in cross-correlations of an image with SNR = α with a virtually noise-free average. Abscissa, SNR of a single image. Ordinate, cross-correlation coefficient *C*.

The specific value of the cross-correlation coefficient is also affected when images exhibiting a low SNR are correlated with an almost noise-free reference. Under these conditions, the correlation coefficient manifests an asymptotic behavior (Fig. 5 ▸). For this particular calculation the value *N* in (10) was set to 1000. The abscissa shows the SNR and the ordinate the cross-correlation coefficient. Its value is always smaller than 1.

Multireference alignments are often used as classification tools. The extent of signal differences between the images determines whether the bias introduced by differences in the SNR of the references will have a significant effect on the outcome. If there are large differences among the signals in the images, the signal differences will dominate the selection process. However, if the differences are small then the bias can be significant. The effect is dominated by an asymptotic behavior, as shown in the curves in Figs. 4 ▸ and 5 ▸. The possible bias diminishes when either the SNR of the images increases or the SNR of the references increases. The effect is obvious in our model calculations, where in one single round of correlations a plurality of images were assigned to the average calculated from the most images, while none of the images were assigned to the averages calculated from 30 or fewer images.

The cross-correlation value can be corrected by multiplication by the inverse of either (10) or (13) and will result in a cross-correlation coefficient of close to 1 if identical motifs exist in the reference and in the image (Fig. 3 ▸ *e*). The correction defined in (10) is implemented in our 3D reference-based projection alignment (Radermacher, 1994 ▸), where the angular distribution of the projections used in the calculation of the 3D reference is often uneven. In this alignment procedure 2D Radon transforms of the projections are cross-correlated with the reference 3D Radon transform. The 3D Radon transform is created by an algorithm that averages the 2D Radon transforms of the projections into a 3D Radon transform. Accordingly, each radial line is an average of multiple radial lines from each of the projections. A counter is maintained indicating how many projection lines contribute to the average in each radial line at any specific angle (φ*_i_*, θ*_j_*). The cross-correlations are carried out by cross-correlation of each radial line in the 2D transform of the projection with each radial line in the 3D transform of the reference. The algorithm as implemented allows the (optional) normalization of each line correlation with the inverse of (10), using the counter of each line for *N*. The Radon inversion of the line-by-line cross-correlations at every angle provides the cross-correlation function. For normalization, the SNR of either the projection or the 2D Radon transform of the projection needs to be known. If the SNR of the image is not known *a priori*, it can be estimated by performing the cross-correlation between a single image and an average image. The best results are obtained when the calculation is carried out in the asymptotic range of (10).

One of the main sources of noise in low-dose electron micrographs is shot noise, which is approximately constant throughout the whole spectrum. Thus, the SNR strongly depends on the image resolution. The signal energy typically weakens towards higher frequencies or better resolutions. Therefore, besides using masks to exclude background noise, low-pass filters are extensively applied in alignment procedures by cross-correlation. Above a certain radius low-pass filters remove more noise energy than signal, thus increasing the SNR. Any SNR value used for correction of the influence of noise on the cross-correlation must be estimated taking into account any mask and band-pass filter applied in the cross-correlation process. One should be careful not to overcompensate for the SNR; the cross-correlation coefficient must not exceed a value of 1. The statistics of the cross-correlation coefficients in the alignment process can be used as a safeguard against overcompensation.

The recently introduced method of projection-based volume alignment (Yu *et al.*, 2013 ▸) for aligning 3D volumes of macromolecular structures could easily take advantage of the theoretical principles described here. In this method, a set of projections with known orientations calculated from a reference volume are aligned with the volumes whose orientations are to be determined. If the reference volume is calculated by averaging the 2D Radon transforms of the projections into a 3D Radon transform, the extracted projections can also provide an index for each radial line that specifies how many projections contributed to each line. A correction for the influence of noise can now be implemented in this algorithm using (13) by making use of the occupancy counters of both the reference 3D Radon transform and the 3D Radon transform of the volumes being aligned.

## 5. Conclusion   

Early literature has described the value of the cross-correlation coefficient when two images with the same noise level are correlated. Here, we extended this equation to include cross-correlations between images and their averages, and between image averages, all obtained from the same original noisy data. The results provide a method to correct for the major influence of noise, and we hope that they will help to increase the awareness of possible bias in multireference alignments and classification.

## Figures and Tables

**Table 1:** Assignments resulting from a multireference correlation process without and with compensation for the SNR Without correction, most images were assigned to the average reference calculated from the larger number of images. With correction using (10) all images were assigned to the correct reference, independent of the number of images used in the average.

Reference	1	2	3	4	5	6	7	8	9	10
No. of images assigned										
Without correction	0	0	0	9	82	99	112	140	247	311
With correction	100	100	100	100	100	100	100	100	100	100
